# Sensing the Stiffness: Cellular Mechano-Sensing at the Implant Interface

**DOI:** 10.3390/cells14141101

**Published:** 2025-07-17

**Authors:** Patricia S. Pardo, Delia Danila, Raja Devesh Kumar Misra, Aladin M. Boriek

**Affiliations:** 1 Section of Pulmonary and Sleep Medicine, Department of Medicine, Baylor College of Medicine, Houston, TX 77030, USA; pardopatriciasusa@gmail.com (P.S.P.); daniladelia@gmail.com (D.D.); 2 Department of Metallurgical, Materialsand Biomedical Engineering, The University of Texas, El Paso, TX 79968, USA; dmisra2@utep.edu

**Keywords:** biomaterials, nanobiotechnology, mechano-sensing, cell stiffness, focal adhesions, hippo signaling, Yes-associated protein (YAP), PDZ-binding domain (TAZ)

## Abstract

In this perspective, we highlight the relevance of the FA-Hippo signaling pathway and its regulation of the Yes-associated protein (YAP) and the transcriptional coactivator with a PDZ-binding domain (TAZ) as main players in the process of implants integration. The modulation and responses of YAP/TAZ triggered by substrate and ECM stiffness are of particular interest in the construction of materials used for medical implants. YAP/TAZ nuclear localization and activity respond to the substrate stiffness by several mechanisms that involve the canonical and non-canonical Hippo signaling and independently of the Hippo cascade. YAP/TAZ regulate the expression of genes involved in several mechanisms of relevance for implant integration such as the proliferation and differentiation of cell precursors and the immune response to the implant. The influence of substrate stiffness on the regulation of the immune response is not completely understood and the progress in this field can contribute to the designing of an adequate implant design. Though the use of nano-biomaterials has been proved to contribute to implant success, the relationship between grain size and stiffness of the material has not been explored in the biomedical field; filling these gaps in the knowledge of biomaterials will highly contribute to the design of biomaterials that could take advantage of the cells sensing and response to the stiffness at the implant interface.

## 1. Introduction

Implants are necessary for the reconstruction of tissue that was severely damaged. They function as a bridge between healthy tissue and the substrate that will drive regeneration in the damaged area. During tissue regeneration, the formation of new tissue is largely influenced by the physicochemical cues of the microenvironment [1].

Most cell types can respond to a wide but limited range of substrate elasticities, and such cells tune their stiffness to that of the substrate material. For this reason, biomaterials with similar stiffness to the damaged tissue render better outcomes. A recent study performed on rabbits with osteotomy of their right tibia showed that the treatment with plates of a titanium alloy with a low elasticity modulus (49.1 GPa) similar to the one of cortical bone (20 GPa) had a greater positive effect on bone formation and bone strength restoration than the treatment with implanted plates of commercially pure titanium (107 GPa) [1].

Understanding the processes involved in cell responses to the substrate’s physical properties is fundamental for substrate patterning and could potentially advance tissue engineering. The integration of an implant occurs in several steps that sequentially involve interaction with the water molecules, adhesion of circulating plasma proteins, the formation of the extracellular matrix, the propagation of the cell precursors, and their proper differentiation into mature tissue. The initiation of the integration occurs at the surface of the implanted material. Firstly, water molecules react with the surface during the first nanoseconds, then a variety of proteins adsorb to the surface during the next few seconds, forming a considerably thick protein layer. In a third stage that lasts a few hours, cells interact with the protein layer. Finally, cell proliferation can take a few days or years, depending on the growth rate of each cell type and the magnitude of the replacement [2].

The ability of cells to respond to external forces and the mechanics of their substrates is determined by the mechanical properties of the cell itself and its ability to respond by generating force. External forces applied to a cell and the resistance that extracellular matrices exert on cell-derived forces also generate signals that direct cell growth, survival, differentiation, and function [3]. Focal adhesion sites are elongated structures that connect clustered integrins to extracellular matrix (ECM) fibrils and to contractile actomyosin stress fiber bundles in the cytoskeleton. FA complexes and the related extracellular matrix adhesions sites act as mechano-sensory ‘devices’, where internal contractile forces or externally applied force can regulate the assembly of the adhesion sites and trigger another mechano-dependent signaling [4,5]. In the context of the implant devices, FA-related mechano-regulated pathways could potentially be responsible for the better biological response of implants.

In this perspective, we emphasize the relevance of the FA-Hippo signaling pathway and its regulation of the Yes-associated protein (YAP) and the transcriptional coactivator with a PDZ-binding domain (TAZ) as the main mechano-signaling pathway that responds to substrate stiffness by controlling the expression of genes involved in several mechanisms of relevance for implant integration. Among them, the ones involved in cytoskeletal re-modeling, cell–ECM interaction and the reprogramming of differentiated cells into tissue-specific cell progenitors are of fundamental relevance for proper implant integration and the healing of damaged tissue.

## 2. Current Advances in the Materials of Medical Metallic Implants: The Benefits of Nano-Structured Materials

As described in the introduction, the integration of an implant occurs in several steps that includes the coverage of the implant with circulating plasma proteins, the formation of ECM, cells proliferation and differentiation into mature tissue.The effectiveness of these processes has been used as parameters to determine the adequacy of various kinds of implants materials [6,7,8,9].

For the load-bearing dental and bone implants, the biomaterial needs to have high fatigue resistance and tensile, compressive, and shear strength. Thus, metallic biomaterials are more suitable for this purpose. Stainless steel and titanium (Ti) alloys are the most widely used for dental implants [10] and orthopedic devices [11]. The strength, corrosion resistance, and their impact on cellular functionality can be enhanced by introducing sub-micron and nanometer-scale microstructural features via Severe Plastic Deformation (SPD) methods [6,12,13,14,15]. Continuous Equal Channel Angular Pressing (C-ECAP), a modern deforming process that produces ultrafine-grained materials [16], alters grain boundary structure and increases the areal surface density of grain boundaries [17]. Misra’s group developed a scanning probe microscopy method that allows the nano-structuring of biomedical-grade austenitic stainless steel with high strength without elongation [6].

The material stiffness can be measured by its modulus of elasticity and should be considered as another relevant mechano-physical characteristic. The biomaterial’s surface must provide mechanical support and maintain the equilibrium of forces that determine cell shape and function. For example, dental implants should be made of a material with a high friction coefficient and a low elasticity modulus. The modulus of elasticity of commercially pure titanium is 100–115 GPa, while the values for different types of bone are 10–30 GPa. Such mechanical incompatibility could potentially provoke “stress shielding”, where the bones are underloaded, which leads to bone resorption and loosening of the implant [18,19]. The development and evaluation of new titanium alloys with low elastic modulus have received major attention from researchers in the field [20,21].

Several studies comparing the behavior of cells seeded on titanium (Ti) implants with different grain sizes demonstrate that the presence of nanoscale features on the surface has a significant effect on how the cells interact with the implant [7,14,15]. The results of these studies have demonstrated that the growth of pre-osteoblasts and other cell types on ultrafine-grain Ti can be increased from 1.3-fold (30% increase) up to 20-fold (1900%) compared to conventional coarse-grain (CG) Ti. These studies have established a positive correlation of grain boundary length with cell attachment and proliferation on ultrafine-grain Ti.

On austenite nano-structure surfaces, proteins adsorbed to the surface of the implant favored the attachment of cells. The high surface coverage of proteins at the nanocrystalline (NC) surface is dramatically different in terms of the self-assembled structure and significantly reduced coverage found on the microcrystalline (MC) surface counterpart [8]. The surface coverage of adsorbed proteins increased from ~20% on the MC to ~85% when the average crystal size decreased from ~22 µm (MC) to ~90 nm (NC).

The analysis of cell morphology and spreading behavior of pre-osteoblasts on the protein-adsorbed surfaces revealed that osteoblasts on the protein-absorbed NC surface exhibited abundant cytoplasmic extensions, higher cell spreading areas, and cell–cell junction formation. The cytoplasmic extensions, formed as thin sheet-like lamellipodia or finger/needle-like protrusions called filopodia, were extensive along the leading edges of the cells on the NC substrate. The presence of lamellipodia and filopodia indicates that the cells are spreading. Such increased organization of cytoskeletal fibers on the NC surface is related to an increased number of crystal boundaries on the NC surface compared to the conventional MC surface [8]. Cell spreading contributes to osteoblast differentiation with increased osteopontin and osteocalcin expression [22]. As the main circulating progenitors in blood are mesenchymal stem cells (MSCs), it is even more relevant that their commitment to different lineages (adipocytes, chondroblasts, or osteoblasts) depends on cell density, cell shape, and the cytoskeleton. Whereas dense, rounded cells are committed to the adipocyte lineage, elongated and spread cells commit to the osteoblast lineage, even before the addition of growth factors necessary for differentiation [23].

Magnesium (Mg) biomaterials and its alloys are also widely used in bone implants due to its biodegradability and mechanical properties close to the bone tissue [24]. One of the most important features of Mg biomaterials is their contribution to bone formation. Mg ions (Mg^2+)^ determine the rate of mineralization and hydroxyapatite growth during bone formation. Moreover, Mg^2+^ channel magnesium trans-porter 1 (MAGT1) controls the influx of Mg^2+^ and promotes bone marrow mesenchymal stem cells differentiation into osteoblasts [25]. Experiments performed on osteoporotic rats subjected to a transversal femoral osteotomy showed thatthe group harboring magnesium-alloy-implants-healed bones had higher bone mass, bone density and load bearing capacity than the group treated with stainless steel implants [26]. The main disadvantage of Mg-based alloys is their low corrosion resistance in the physiological media; corrosion changes the mechanical properties of the Mg biomaterial and eventually may result in implant failure [24].

Among metallic biomaterials, tantalum (Ta) is one of the most promising for orthopedic bone applications as long as the costs of manufacturing are reduced. Although the metal Ta is bioinert the porous Ta exhibits excellent osseointegration and osteo-conductivity. In addition, Ta possesses excellent biocompatibility, mechanical ductility and corrosion resistance [27]. Its high affinity for oxygen results in a surface oxide layer (Ta2O5) that resembles bone apatite. Because the elastic modulus of porous Ta is equivalent to that of the human cancellous bone, the stress load is evenly distributed on a porous Ta implant, thereby minimizing the risk of dissolution around the prosthesis and implant failure caused by stress shielding. Porous Ta scaffolds facilitate the attachment, proliferation, differentiation, and mineralization of osteoblasts, leading to better osteogenesis and osteointegration in vivo [28,29].

Synthetic polymeric materials have been used since the 1980s as implantable materials or as a coating on medical implants to enhance their properties [30]. They include ceramics, polymeric biomaterials and composites.

Ceramic materials are widely used in orthopedic surgery to repair or replace bone tissue as their chemical properties resemble bone matrix. Ceramics include bioinert materials such as alumina and zirconia and bioactive materials such as hydroxyapatite, calcium phosphates and bio-glass. Bioactive materials are the biomaterials that stimulate a biological response and can modify the surrounding tissue biologically. They can be osteo-conductive or osteo-productive [31]. Osteo-conductive materials such as hydroxyapatite and other calcium phosphates bind to the bone and enhance bone growth along their surface, they degrade during the healing process and are replaced by new tissue. Bioactive glasses are osteo-productive which means that they can favor the growth of new bone away of the bone/implant interface. The most common bio-glass is 45S5 composed of 45.5% SiO_2_, 24.5% Na_2_O, 25.5% CaO, and 6% P_2_O_5_. It is used in clinics for the treatment of periodontal diseases and as a bone filling material, i.e., for the replacement of damaged ear bones, which helps to restore hearing [32].

Polymeric biomaterials can be designed accordingly to the specific applications by modifying their chemical composition, molecular weight and crystallinity. This offers the possibility of modifying their physical and chemical properties by the appropriate designing of the polymer functional groups. Their hydrophobic and hydrophilic nature could be tailored to avoid immunogenicity or osteo-conductivity. Poly (L-Lactic Acid) (PLLA), Poly (Lactic-Co-Glycolic Acid) (PLGA) and Poly(ԑ-Caprolactone) (PCL) are synthetic polymers used in the fabrication of implants and often used for coating metallic implants for better osseointegration [33]. The rate of degradation of PLGA can be adjusted by altering the ratio of lactic acid and glycolic acid which makes them suitable for the sustained release of drugs and growth factors at the site of the damaged tissue [34].

As is the case for metallic biomaterials, the non-metallic biomaterials enhance their effectiveness in the last stages of the implant integration when they hold nano-structure features [35,36]. Polymeric nanomaterials provide structural support and a surface that facilitates cell migration and activity. In addition, when the osseointegration is complete, they are resorbed, which avoids the complications related to biomaterials that are not degradable.

Synthetic nanomaterials, like bioactive ceramics such as hydroxyapatite (HA) and tricalcium phosphate (TCP), and composites like poly-lactic acid (PLA) and poly-glycolic acid (PGA) are presently recommended as scaffolding substances for managing osseous deformities due to its capability to supply enhanced structural strength. External treatment with growth factors such as bone sialoproteins (BSP) and bone morphogenic proteins (BMP) might enhance the capacity of these nano-structured biomaterials to attain effective osseointegration [36].

## 3. Role of Mechano-Signaling Pathways in Sensing the Stiffness of the Cell–Substrate Surface

An equilibrium of forces between cells and the extracellular matrix maintains cell shape and function [37,38]. The tensional state and organization of the cytoskeleton reflect the local pattern of geometrical and mechanical strains associated with the position of the cell within the tissue architecture and surrounding stromal composition [39,40]. These inputs include the forces emanating from the topology and rigidity of the extracellular matrix (ECM) [41]. In the context of an implant, cell precursors and immune cells are the main subjects of these mechanical cues.

The cells can detect changes in ECM mechanics by the components of the ECM–integrin–cytoskeleton linkage localized in the FA complexes [42]. On soft substrates, integrins are dispersed along the cell membrane and F-actin is not highly polymerized; alternatively, on rigid substrates, integrins cluster and interact with focal adhesion proteins such as vinculin, talin, and focal adhesion kinase (FAK). Similarly, cells react to the mechanical stress exerted from the substrate by activating RhoA and Rho-associated kinase (ROCK), which promotes the assembly of an acto–myosin contractile network, the so-called “stress fibers”, and FA assembly. FA complexes are connected to the cytoplasm and ECM through the clustered integrins, and on their cytoplasmic side they connect to the stress fibers [43,44].

Yes-associated protein (YAP) and transcriptional coactivator with a PDZ-binding domain (TAZ) are responsible for the conversion of these signals into changes in gene expression. YAP and TAZ shuttle from the cytoplasm to the nucleus where they function as co-transcription factors that activate Transcriptional Enhancer factor/Activator of transcription (TEA) domain family members (TEAD) [45,46].

YAP/TAZ activity is under direct control of the cell shape as dictated by the cytoskeletal structure and converts such inputs into gene expression and context-dependent biological responses [44,47], including proliferation, cell plasticity, and stemness essential for tissue regeneration.

**Figure 1:**
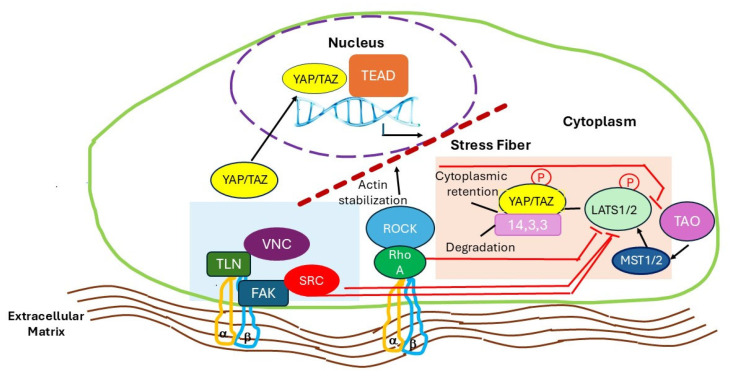
The HIPPO core pathway and its regulation by focal adhesions.The core of the Hippo pathway (light orange block) is a kinase cascade formed by the serine/threonine kinases MST1/2 and LATS1/2, and the transcription coactivators of TEAD, YAP and TAZ. When the Hippo pathway is activated, TAO phosphorylates MST1/2 which upon activation phosphorylate LATS1/2. Then, activated LATS1/2 phosphorylates and inhibits YAP and TAZ, preventing them from translocating into the nucleus to interact with TEAD transcription factors. Thus, activation of the Hippo kinase cascade results in inhibition of the transcriptional output module. The nuclear localization and activation of YAP/TAZ relies on the formation of stress fibers under the control of RhoA and ROCK that inhibits TAO and the interaction of integrins (α and β) embedded in the extracellular matrix (ECM) with the focal adhesion complexes (highlighted in light green). Integrin binding to talin (TL) and vinculin (VCN) activates focal adhesion kinase (FAK)-steroid receptor coactivator (SRC) which repress YAP/TAZ phosphorylation by LATS1/2. Activation mechanisms are shown as black arrows, and inhibitory mechanisms are indicated in red capped lines.

YAP and TAZ shuttling is regulated by the Hippo kinase cascade (Figure 1). In the absence of the highly polymerized actin, the Hippo pathway core kinases such as Mammalian Ste-20 like kinase 1 and 2 (MST1/2) are activated by phosphorylation by Thousand and one kinases (TAOKs) which interestingly regulates actin depolymerization [48].

Upon activation, MST1/2 phosphorylate the C-terminal hydrophobic motif of Large Tumor Suppressor kinase 1 and 2 (LATS1/2). Active LATS1/2 phosphorylates and promotes the cytoplasmic retention of YAP and TAZ via a 14-3-3 interaction and the degradation of YAP/TAZ, ultimately preventing TEAD-mediated transcription [49]. Therefore, the activation of the upstream kinase module ultimately results in inhibition of the transcriptional output module [50]. On the other side, the stability of actin cytoskeleton and cell tension triggers the activation of YAP and TAZ by inhibiting TAOK through RhoA/ROCK and FAK [48]. By binding to cell- and context-specific transcription factors, YAP/TAZ contributes to ECM remodeling [51,52,53,54]. YAP/TAZ nuclear activity is correlated to the stability of the actin cytoskeleton and cell tension, as controlled by myosin light chain II and Rho GTPase pathways [55,56,57]. Two upstream signaling pathways contribute to the nuclear localization and activity of YAP/TAZ: (1) Rho/GTPases control the formation of actin bundles and stress fibers in response to the cell spreading over the ECM [34,54] and (2) Integrin-FA signaling targets the steroid receptor coactivator SRC protein kinases which repress YAP and TAZ phosphorylation by LATS [44]. YAP/TAZ transcriptional activity is modulated along the Rho/Rock axis as cells spread over the surrounding ECM [56,58] to determine FA–cytoskeleton remodeling. Thus, YAP/TAZ act as regulators of ECM mechanics [42] and the main regulator of cell–ECM interaction. YAP/TAZ activation can also endow cell plasticity, reprogramming primary, genetically normal differentiated cells into their corresponding tissue-specific stem progenitors [59]. In mammalian skeletal stem cells (SSCs), the zinc finger transcription factors Snail and Slug promote stem cell proliferation and differentiation by forming complexes with YAP/TAZ; the Snail-Slug/YAP/TAZ and Snail-Slug/TAZ/RUNX activate target genes that control SSC proliferation and osteogenesis [60]. Furthermore, YAP/TAZ promote osteogenic differentiation through the alternative Wnt-YAP/TAZ signaling axis [61].

However, the activation of YAP/TAZ can negatively impact the stability of the implant during the healing process through its effects on the immune response to the implanted material. Despite their relative low abundance in immune cells compared to other cell types, YAP1 expression increases upon differentiation of monocytes to adherent macrophages and macrophages undergoing pro-inflammatory [62] and reparative programs [63]. Macrophages play a pivotal role in the immunological cascade activated in response to biomedical implants, which predetermine acceptance or rejection of implants by the host via pro- and anti-inflammatory polarization states, M1 and M2, respectively. Mey et al. [64] found that bone marrow-derived macrophages (BMDM) are ‘primed’ to a pro-inflammatory M1 phenotype on stiff substrates, as opposed to an anti-inflammatory M2 phenotype on soft and medium stiffness substrates. The matrix stiffening increases the expression of the stretch activated ion channel Piezo1, which leads to the activation of YAP, favoring M1 polarization and suppressing M2 polarization. Moreover, the treatment with a YAP inhibitor, successfully induced macrophage re-polarization to the M2 state within the implant site microenvironment, which in turn promoted implant osseointegration. Whereas YAP/TAZ activation promotes tissue re-generation in non-immune cells by inducing genes involved in cells proliferation, the activation of YAP/TAZ in immune cells leads to implant failure by favoring inflammation, impairing healing and promoting fibrosis.

## 4. Control of YAP/TAZ Subcellular Localization Mediated by Mechanical Cues

In Section 3, we described shuttling of YAP/TAZ during cell spreading by changes in YAP/TAZ state of phosphorylation driven by the Hippo pathway that is triggered by the sensing of ECM mechanical cues through integrin-FA signaling and the perception of the cytoskeleton stability.

**Figure 2:**
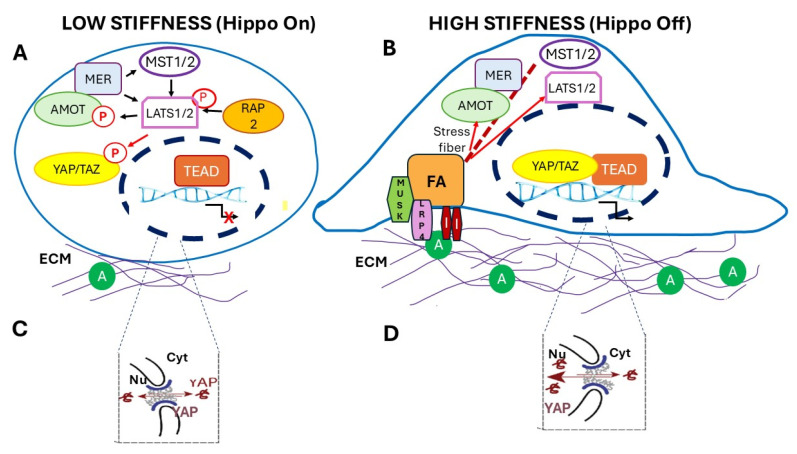
Regulation of YAP/TAZ subcellular localization by substrate/ECM stiffness. (**A**) In cells attached to low-stiffness substrates and/or low-stiffness ECM, low polymerization of F-actin results in release of AMOT from F-actin. AMOT binds and activates Merlin (MER) and the AMOT/Merlin complex activates LATS1/2 ensuring the cytoplasmic retention of YAP and TAZ. RAP2 (RAS-related GTPase) also activates LATS1/2 and determines inhibition of YAP/TAZ by cytoplasmic retention. (**B**) In cells on high stiffness substrate/ECM, AMOT is bound to highly polymerized F-actin (Stress fibers) and Merlin remains in its inactive conformation. High ECM stiffness also promotes high expression of Agrin (**A**) that, bound to LRP4, activates the muscle-specific kinase (MUSK). MUSK inactivates LATS1/2 and shuts down the activity of Merlin leading to nuclear localization of YAP/TAZ. (**C**) In cells on soft substrates, the connection between the ECM and the cytoskeleton is loose and no force is transmitted to the nuclei; nuclei are round-shaped, and the nuclear pores’ diameter does not allow passive entry of YAP/TAZ. (**D**) Rigid substrates transmit forces through the cytoskeleton to the nuclear membrane, nuclei are flattened, and the nuclear pores’ diameter is increased allowing the passive import of YAP/TAZ. (**C**,**D**) were modified from Elosegui-Artola et al. (2017) [68]. Black arrows represent the activation of the target, red arrows or red crosses indicate inhibition.

The modulation and responses of YAP/TAZ triggered by substrate and ECM stiffness are of particular interest in the construction of materials used for medical implants. ECM/substrate stiffness determines the localization and activity of YAP/TAZ in response to the state of F-actin polymerization and cytoskeletal structure modification. In cells with low ECM stiffness, there is low F-actin polymerization, angiomotin (AMOT) is released from F-actin and binds to MERLIN, and MERLIN binds to YAP/TAZ. These proteins in a complex activate the Hippo kinase cascade and determine YAP/TAZ cytoplasmic retention [65]. The RAS-related GTPase RAP2 also contributes to LATS1/2 activation, cytoplasmic retention of YAP/TAZ, and inhibition of the YAP/TAZ-dependent transcriptional activation [66] (Figure 2A). In turn, in cells with high ECM stiffness, highly polymerized F-actin forms stress fibers, and AMOT and MERLIN are bound to F-actin in an inactive state. High ECM stiffness also increases the expression of Agrin, an ECM proteoglycan that binds to low-density lipoprotein receptor-related protein (LRP4). Agrin bound to LRP4 activates the muscle-specific kinase (MUSK) that shuts down the activity of Merlin and LATS1/2, leading to YAP/TAZ nuclear localization [67] (Figure 2B).

Although many upstream signals regulate YAP/TAZ activity through the Hippo kinase cascade, an increasing number of studies have uncovered other mechanisms that regulate YAP/TAZ subcellular localization, stability, and activity independently of the Hippo kinase cascade. YAP/TAZ post-translational modifications, such as phosphorylation, ubiquitination and deubiquitylation, sumoylation, methylation, or O-GlcNAcylation, as well as interactions with several binding partners, can modulate YAP/TAZ localization and activity in response to various stimuli in a Hippo-independent manner [69].

Nuclear localization of YAP can be triggered independently of the Hippo pathway (Figure 2C,D). Elosegui-Artola et al. [68] demonstrated that cells respond to ECM rigidity. In cells growing on soft substrates, YAP localizes in the cytoplasm. However, when the rigidity of the substrate has an elastic modulus over 5 kPa, YAP translocates to the nuclei in response to the force applied to the nucleus. On stiff substrates, formed stress fibers and focal adhesions transmit the force from the ECM to the nuclear cytoskeleton, flattening the nucleus and stretching the nuclear pores, which allows the import of YAP and other proteins with low weight or stability. In the nucleus, the SWI/SNF chromatin remodeling complex interacts with YAP/TAZ through ARIDIA and inhibits its association with TEAD and, therefore, YAP/TAZ transcriptional activity. Under mechanical stress, nuclear F-actin binds to the ARIDIA-SWI/SNF complex and prevents its association with YAP/TAZ; thus, the YAP/TAZ interaction with TEAD and transcriptional activity are restored [70].

## 5. Discussion and Conclusions

Implants are necessary for the reconstruction of tissue that was severely damaged. They function as a bridge between healthy tissue and the substrate that will drive regeneration in the damaged area. During tissue regeneration, the formation of new tissue is largely influenced by the physicochemical cues of the microenvironment. Cells are capable of sensing the mechanical properties and the topography of the substrate; they convert those mechanical signals into mechanical and biochemical responses in a way that cycles of mechano-sensing and bio/mechano-responses modify their behavior and the properties of the environment [39,71].

At the core of this matter, YAP/TAZ emerged as two highly related transcriptional regulators that sense how cells perceive themselves and their tissue environment and communicate with it [72]. YAP/TAZ activity is under direct control of the cell shape dictated by the cytoskeletal structure [44]. The tensional state and organization of the cytoskeleton reflect the local pattern of geometrical and mechanical strains associated with the position of the cell within the tissue architecture and surrounding stromal composition [39,40]. These inputs include the forces emanating from the cells’ attachment to other cells and from the topology and rigidity of the extracellular matrix (ECM) [41]. YAP/TAZ convert such inputs into gene expression and context-dependent biological responses [44,47] including proliferation, cell plasticity, and stemness essential for tissue regeneration. YAP/TAZ activation can endow cell plasticity, reprogramming primary and genetically normal differentiated cells into their corresponding tissue-specific stem or progenitors [60]. Further, YAP/TAZ promote osteogenic differentiation through the alternative Wnt-YAP/TAZ signaling axis [61].

Studies on the activation of YAP/TAZ and Hippo signaling during the steps of the implant integration may highly contribute to the understanding of this process and the evolution in the design of adequate implants. In the in vitro models of implant integration, knockdown of YAP or TAZ will allow us to determine their function in this process and the use of cells containing YAP or TAZ reporters would be useful to determine their transcription activity during the steps of implant integration. Whole-animal knockdown for YAP and TAZ are not viable. Two mice models with osteocyte-specific inducible knockdown of YAP and TAZ had been reported to bear lower bone mass, altered mechanical properties and impaired bone matrix remodeling [73,74]. It will be interesting to investigate how these mice will respond to treatment with implants after bone injury.

The studies on the effects of grain size on several biomaterials have shown the benefits of nano-structured biomaterials on cells adhesion, spreading and morphology. However, there is not a clear relationship between the changes from micro to nano-structured materials with changes in stiffness. Abazari et al. [75] analyzed five theories that try to quantify the effect of size structure on the mechanical properties of the materials used for micro and nanomechanical systems. These authors compared Grain Boundary Theory (GBT), Surface Stress Theory (SST), Residual Stress Theory (RST), Couple Stress Theory (CST) and Surface Elasticity Theory (SET) with experimental data and proposed a simplified model combination of CST and SET that can be used to predict the mechanical properties at the nanoscale [76]. Similar studies performed in biomaterials for medical implants might be useful in the fabrication of new nanomaterials with a desired stiffness considering that the surface stiffness of a material similar to the physiological tissue stiffness was found to be the most robust input for nuclear YAP/TAZ translocation [76].

### Future Directions

The permanent activation of YAP/TAZ might lead to tissue overgrowth and the consequent loosening of the implant. In order to avoid tissue overgrowth after the implant integration, the use of YAP/TAZ inhibitors might be a useful approach. Several strategies are being pursued to inhibit YAP/TAZ function, including the development of small molecule and RNAi-based inhibitors; some of them are now under clinical trials for cancer therapy [77,78] whole-body inhibition of YAP/TAZ may have serious side effects, it would be ideal to develop effective means to manipulate the Hippo pathway and YAP/TAZ activity in both a tissue specific and transient manner. In this sense, the implant surface might be used to allow a localized delivery of the inhibitor by combining the implant with a nanoparticle slow drug delivery system. There is no precedent for the concourse of these tools of nanomedicine combined, but undoubtedly, addressing these challenges could contribute to the advancements in the fabrication process of biomaterials with the proper structural features that can provide the optimal mechanical and biochemical environment to achieve the desired cell responses in vivo.

## Abbreviations

The following abbreviations are used in this manuscript:

ECM	Extracellular Matrix
MC	microcrystalline
NC	nanocrystalline
YAP	Yes-associated protein
TAP	Transcription co-activator with a PDZ domain
Ti	Titanium
E-CAP	Continuos Equal Channel Angular Pressing
SVD	Severe Plastic Deformation
GBT	Grain Boundary Theory
SST	Surface Stress Theory
RST	Residual Stress Theory
CST	Couple Stress Theory
SET	Surface Elasticity Theory
